# First records of Gastrotricha from South Africa, with description of a new species of *Halichaetonotus* (Chaetonotida, Chaetonotidae)

**DOI:** 10.3897/zookeys.142.2036

**Published:** 2011-10-31

**Authors:** M. Antonio Todaro, Matteo Dal Zotto, Sarah J. Bownes, Renzo Perissinotto

**Affiliations:** 1 Dipartimento di Biologia, Università di Modena e Reggio Emilia, via Campi, 231/D, I-41125 Modena, Italy; 2 School of Biological & Conservation Sciences, University of KwaZulu-Natal, Westville Campus, Private Bag X54001, Durban 4000, South Africa

**Keywords:** Gastrotricha, meiofauna, new species, South Africa, St. Lucia Estuary, iSimangaliso Wetland Park

## Abstract

During a survey of the biota of the St. Lucia Estuary in the iSimangaliso Wetland Park, South Africa, a number of Gastrotricha were found among samples of meiofauna. Fresh, marine sediment yielded several specimens belonging to a total of seven species. Of these, two are already known from other regions (i.e., *Dactylopodola australiensis* and *Heteroxenotrichula squamosa*), one is described as new to science (*Halichaetonotus sanctaeluciae* sp. n.), while the remaining four (*Pseudostomella* sp., *Halichaetonotus* sp.1, *Halichaetonotus* sp. 2, *Xenotrichula* sp.) require further collections and analysis, in order to establish the extent of their affiliation to species already described. General appearance, shape of hydrofoil scale and the occurrence of three long spines on the dorsal side make the new species most closely related to *Halichaetonotus australis* and *Halichaetonotus marivagus*. The key differences from these taxa and between *Halichaetonotus sanctaeluciae* sp. n. and *Halichaetonotus aculifer* are discussed.

## Introduction

Marine Gastrotricha from Africa are poorly known. Apart from the pioneering research carried out in Algeria by d’Hondt (1974) and in Somalia by (Valbonesi and Luporini (1984, 1987), only the study of Hummon (2011) has been undertaken recently in that region. The latter work includes records of marine gastrotrichs from the Mediterranean and Red Sea coasts of Egypt. In addition, scattered records of species can be found for Tunisia (e.g., Westheide 1972; Todaro et al. 2011) and Kenya (see Balsamo et al. 1992, p. 496). *Macrodasys africanus*, described long ago from Namibia, is the only gastrotrich species known from southern Africa (Remane 1950).

This study was initiated after several gastrotrich specimens were recovered from formalin-fixed samples collected during an ongoing investigation of the biodiversity and ecology of meiofauna inhabiting the St Lucia Estuary (KwaZulu-Natal, South Africa). As identification to species of soft-bodied meiofaunal taxa, like Gastrotricha, is best achieved when the taxonomic characteristics are observed on fresh specimens, the collection of a series of samples was undertaken for a dedicated *in vivo* analysis.

It should be highlighted that a census of the biota populating this area bears special relevance, as the St Lucia Estuary is the largest estuarine lake in Africa, a Ramsar Wetland of International Importance and a crucial ecosystem within the iSimangaliso (formerly Greater St Lucia) Wetland Park, South Africa’s first UNESCO World Heritage Site (Taylor 2006).

## Methods

All samples containing gastrotrichs were collected from St Lucia beach (28°23'S; 32°25'E), on the ocean side of the sand berm currently closing the estuary. Sediment samples were collected subtidally (about 40 cm water depth) over a neap tide and spring low tide on 22 February and 7 October 2010, respectively. On each occasion, several samples were collected by gently shovelling 600 ml plastic jars through the top 5–10 cm of sediment until full. Within 1–2 days of collection, samples were sent to Modena (Italy) via courier service. In the lab, gastrotrichs were extracted from the sediment by the narcotization-decantation technique, using an isosmotic (7%) magnesium chloride solution; the fauna-containing supernatant was then poured directly into a 5 cm diameter Petri dish and scanned for specimens under a Wild M8 dissecting microscope, set at 50x magnification (Todaro and Hummon 2008). For taxonomic surveying, the gastrotrichs were removed with a micropipette from the Petri dish, fresh-mounted on slides and observed using a Nikon Eclipse 90i microscope, equipped with Differential Interference Contrast optics (Nomarski) and a DS-5M Nikon digital camera. During the observation, animals were measured with the Nikon ACT-2U software. The description of the new species follows the convention of Hummon et al. (1992, 1993), whereas the position of morphological characters along the body are given in percentage units (U) of total body length measured from anterior to posterior end.

Granulometric analysis of the substrata was carried out according to Todaro et al. (2006). Mean grain size, sorting coefficient, kurtosis, and skewness were calculated by a computerized program based on the equation of Seward-Thompson and Hail (1973).

Abbreviations are as follows: TL, total body length; PhL, pharynx length; FuL, furca length; PhIJ, pharyngeointestinal junction; TbA, adhesive tubes of the anterior series; TbL, adhesive tubes of the lateral series; TbP, adhesive tubes of the posterior series.

## Results

Altogether, 24 samples collected on four different occasions were analysed. Gastrotrichs were only found in sandy material collected in February and October 2010 (Table 1). In total, seven species belonging to five genera and four families representing both orders Macrodasyida (2 spp) and Chaetonotida (5 spp) were found. Two species were identified as known taxa (*Dactylopodola australiensis* and *Heteroxenotrichula squamosa*), one is described here as new to science (*Halichaetonotus sanctaeluciae* sp. n.), while for the remaining four taxa the data gathered so far are not sufficient to exclude their affiliation to species already described. They have, therefore, been provisionally named as follows: *Pseudostomella* sp., *Halichaetonotus* sp. 1; *Halichaetonotus* sp. 2 and *Xenotrichula* sp. (Table 1).

**Table 1.** Gastrotrich taxa found at St. Lucia beach and granulometric characteristics of the microhabitat at the time of sampling during 2010.

**Taxon**	**Sampling date**	
	**22 February**	**7 October**
*Dactylopodola australiensis*	-	+
*Pseudostomella* sp.	+	-
*Halichaetonotus sanctaeluciae* sp. n.	+	+
*Halichaetonotus* sp. 1	+	-
*Halichaetonotus* sp. 2	+	-
*Heteroxenotrichula squamosa*	+	-
*Xenotrichula* sp.	+	-
**Granulometric parameters**		
Mean particle size (phi)	1.95	1.62
Sorting (phi)	0.80	0.77
Kurtosis	3.58	2.55
Skewness	-0.99	-0.10

+, species present; -, species absent.

## Taxonomic account

### Order Macrodasyida Remane, 1925 [Rao & Clausen, 1970]. Family Dactylopodolidae Strand, 1929. Genus Dactylopodola Strand, 1929

#### Dactylopodola australiensis

Hochberg, 2003

http://species-id.net/wiki/Dactylopodola_australiensis

Fig. 1A

##### Material.

1 specimen, South Africa, KwaZulu-Natal, St Lucia beach, 7 October 2010, SJ Bownes legit.

##### Morphometry.

TL, 319 µm; PhL, 92 µm; PhIJ at U26; TbA, 4 per side; TbL, 1+1+1+2 per side; TbP, 5 per side; Ocellar granules absent.

##### Remarks.

the single specimen found is a young adult at the male phase. Among the nine species of *Dactylopodola* described so far (Hummon and Todaro 2010), the body shape of the specimen from South Africa most resembles *Dactylopodola australiensis* Hochberg, 2003, *Dactylopodola indica* (Rao & Ganapati, 1968), *Dactylopodola mesotyphle* Hummon, Todaro, Tongiorgi & Balsamo, 1998 and *Dactylopodola typhle* (Remane, 1927). By virtue of its body size (considering the age), number and arrangement of the adhesive tubes our specimen best approaches the morphometric traits of *Dactylopodola indica* and, especially, of *Dactylopodola australiensis*. While *Dactylopodola indica* is reported (Rao and Ganapati 1968) to have only 2 TbA and 4 TbP per side (vs 4 and 5, respectively), *Dactylopodola australiensis* seems to differ from the South African specimen solely in the length of the pharynx (131 vs 92 µm) and in the position of the pharyngeo-intestinal junction (U34-U35 vs U26) (see Hochberg 2003); it is possible that dissimilarities are due to the early age of the African specimen.

**Figure 1.**
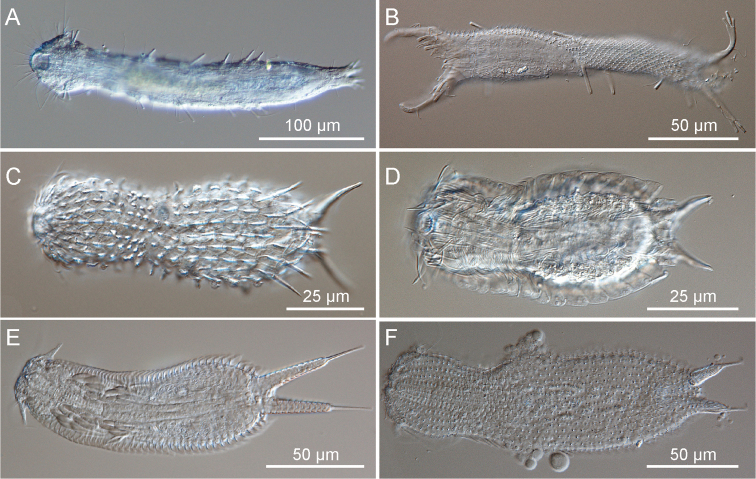
Gastrotricha from St. Lucia beach, South Africa DIC photomicrographs. **A–B** Macrodasyida, **C-F** Chaetonotida. A, *Dactylopodola australiensis*, ventral view B, *Pseudostomella* sp., twisted juvenile **C** *Halichaetonotus* sp.1, dorsal view **D** *Halichaetonotus* sp. 2, ventral view **E** *Heteroxenotrichula squamosa*, ventral view **F** *Xenotrichula* sp., dorsal view.

### Family Thaumastodermatidae Remane, 1927. Subfamily Thaumastodermatinae Remane, 1927. Genus *Pseudostomella* Swedmark, 1956

#### Pseudostomella sp.

http://species-id.net/wiki/Pseudostomella

Fig. 1B

##### Material.

1 juvenile specimen, South Africa, KwaZulu-Natal, St Lucia beach, 22 February 2010, NAF Miranda legit.

##### Morphometry.

TL, 208.6 µm; PhL, 92 µm; PhIJ at U26; oral palps, 36.4 µm in length, showing 5 dorsal and 6 ventral papillae; cuticular covering made up of relatively large tetrancres; TbA, 3 per side; TbL, 5 per side; TbP, 4 per side, 3 at the end of the caudal pedicle and 1 near its base (inner side).

##### Remarks.

The anatomical traits of the animal from South Africa do not seem to match those of any other known species of *Pseudostomella*.However, specimen was a juvenile (i.e., not reproductively mature) and so could not be fully described. Should an adult be found in the future, useful comparisons could be restricted to species bearing a cuticular covering made up of tetrancres and relatively long caudal pedicles.

### Order Chaetonotida Remane, 1925 [Rao & Clausen, 1970]. Suborder Paucitubulatina d’Hondt, 1971. Family Chaetonotidae Gosse, 1864. Genus *Halichaetonotus* Remane, 1936

#### Halichaetonotus sanctaeluciae sp. n.

urn:lsid:zoobank.org:act:61F9C96F-6E24-493A-9CE8-7AC2F06F2CF3

http://species-id.net/wiki/Halichaetonotus_sanctaeluciae

Figs 2 3

##### Type locality.

South Africa, KwaZulu-Natal, St. Lucia beach (28°23'S; 32°25'E); among medium, moderately siliceous grains on a high-energy sandy beach at mid-tide level.

##### Type specimens.

Holotype, the 146.5 μm long adult specimen shown in Figure 3.

##### Material examined.

Four specimens, two adults (including the holotype) plus one subadult collected on 22 February 2010 (NAF Miranda legit) and 1 adult collected on 7 October 2010 (SJ Bownes legit)

##### Diagnosis.

Medium-sized *Halichaetonotus* (LT to 146.6 μm), head, neck and trunk well defined; head rounded, lacking hypostomion but with a small cephalion; medium-long furca projecting from the posterior of the trunk. Body enveloped by 15 columns (7 dorsal, 2 lateral + 2 ventrolateral hydrofoil scales, 2+2 ventral small scales) of alternating keeled scales each with 17–19 scales. Scales, round on head and neck becoming oval to semi-elliptical on the trunk; in general, keel extending beyond the edge of the scales as short spiny processes; on three posterior scales, one median and two lateral, keels forming long and robust spines extending beyond end of trunk. Two small spiny scales on dorsal and several keeled scales on ventral base of furca. Laterally and ventrolaterally, 2+2 columns of hydrofoil scales of varying length; ventrally, 2+2 additional columns of smaller scales; locomotory cilia arranged in two longitudinal bands, interciliary ventral field naked except for two pairs of perianal ovoid keeled/spiny scales. Almost circular mouth opening into cylindrical pharynx with 2 teeth, then sack-like intestine and terminal ventral anus. All specimens parthenogenetic, sometimes with single large egg in position dorsal to mid intestine.

##### Etymology.

The specific name alludes to the geographic locality where the new species has been found.

##### Description.

The description is mainly based on an adult specimen, 146.5 μm in total length. Head rounded, slightly elongated along anterior/posterior axis, bearing a shallow cephalion but no pleural lobes or visible hypostomion; neck narrower than head, trunk sac-like, terminating in a furcate caudum. Body widths at the head/neck/trunk/caudum are 31/22.5/34.5/22 μm, at U11/27/58/81, respectively. Caudum of medium length (26.6 μm), paired laterally divergent adhesive tubes (20 μm in length) with a slightly swollen base (6.5 μm), covered by scales.

*Cuticular armature.* Head, neck, and trunk covered dorsally and lateroventrally by alternating columns (7 dorsal, 1+1 lateral and 1+1 ventrolateral hydrofoil, 2+2 ventral) of 17–19 keeled scales, barely overlapping. On dorsal side, head and neck scales are round (3–5 μm in diameter), while trunk scales are oval to semi-elliptical (9.5 × 5.5 - 12.7 × 6.6 μm). In general, keel on dorsal scales extends beyond the edge of scales as short spiny process; however, on two lateral and one median trunk scales, at U63 and U71, respectively, keels form robust, very long spines projecting 26 μm beyond scales. On posterior trunk region are two oval, double keeled scales (5 × 4 μm) each anchoring a sensorial bristle at U79.5 and a couple of oval spiny scales (4 × 3.5 μm) bearing spines (4–5 μm long) protruding into the furcal indentation. Lateral and ventrolateral spines of hydrofoil scales bearing flattened lamellae, most of which taper into a long hairy process; lamellae bearing spines of the lateral scales are longer than related ventrolateral ones (up to 25 vs up to 19), while lamellae of a column are longest at mid trunk. On ventral side, up to five keeled scales, 3–4 μm long, cover the fleshy portion of each furcal branch; the interciliary field appears naked except for two pairs of oval keeled scales in the perianal region; scales of anterior pair are larger (9.5 × 4.5 μm) than posterior ones (6.0 × 3.5 μm).

*Ventral ciliation.* paired longitudinal bands extending from U03 to approximately U77; each broadly club-shaped anteriorly, but narrowing considerably from the posterior pharyngeal region; bands approach each other immediately behind the mouth, but remain separate throughout their entire length; individual cilia are about 11 μm in length.

*Digestive tract:*Mouth of medium size (ca.6 μm in diameter), projecting very slightly ventrally and leading progressively into a 32 μm long pharynx; pharynx muscular, roughly cylindrical (8 μm in diameter), showing a bulb anteriorly (12 μm in diameter); two cuticular teeth are visible within the bulb; pharynx connected to sack-like intestine at pharyngeo-intestinal junction at U25; intestine straight, narrowing posteriorly, anus ventral at U77.

*Reproductive tract*. Three specimens were in parthenogenic phase, two of which with a large egg filling much of the trunk.

**Figure 2.**
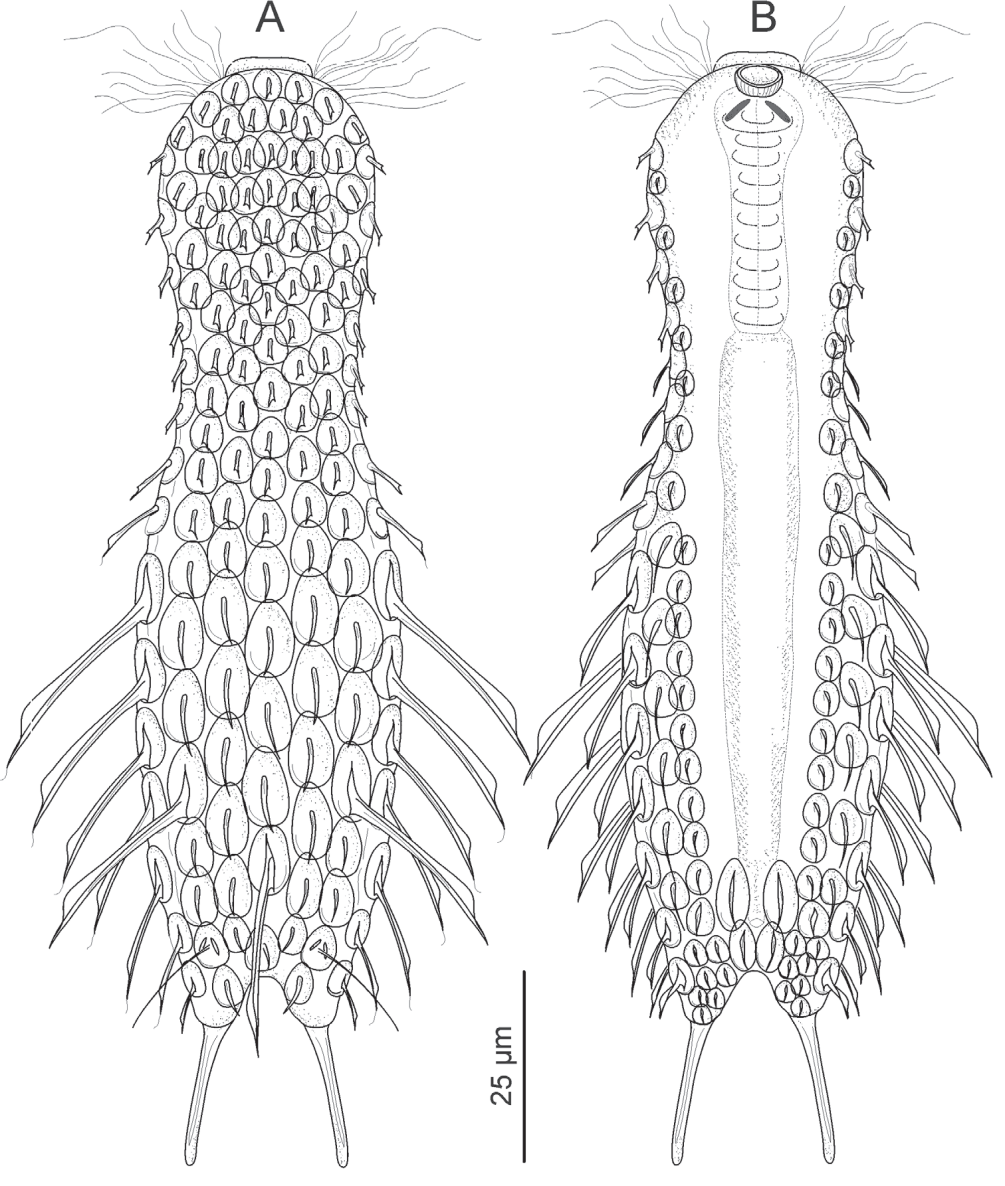
Gastrotricha from St. Lucia beach, South Africa. *Halichaetonotus sanctaeluciae* sp. n., schematic drawings **A** dorsal view **B** ventral view (locomotor cilia omitted).

**Figure 3.**
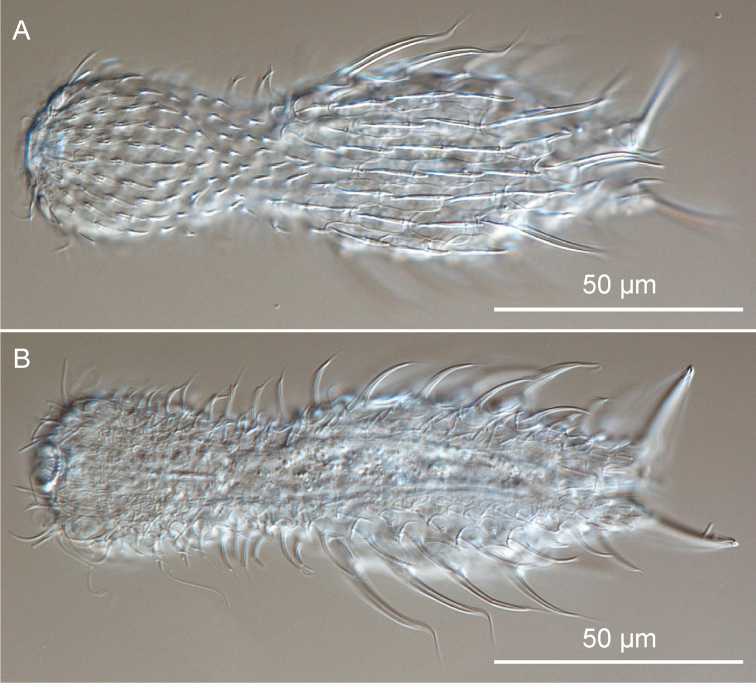
Gastrotricha from St. Lucia beach, South Africa. *Halichaetonotus sanctaeluciae* sp. n., habitus **A** dorsal view **B** ventral view. DIC photomicrographs.

##### Taxonomic affinities.

Highly variable cuticular armature distinguishes the 30 species of *Halichaetonotus* described so far (Hummon and Todaro 2010, Hummon 2010). The new species most closely resembles *Halichaetonotus marivagus*, Balsamo, Todaro & Tongiorgi, 1992, and *Halichaetonotus australis* Nichols & Todaro, 2005, in that all three species are characterised by three dorsal spines close to the posterior end of the trunk. Spines are longest in *Halichaetonotus australis* (up to 46 µm), intermediate in *Halichaetonotus sanctaeluciae* sp. n. (up to 26 µm) and shortest in *Halichaetonotus marivagus* (up to 15 µm).

*Halichaetonotus marivagus* known from the Mediterranean, can easily be distinguished from the new species also on the basis of its wide hypostomion, which is absent in *Halichaetonotus sanctaeluciae* sp. n., and for exhibiting a large cephalion that covers much of the dorsal side of its head (Balsamo et al. 1992).

*Halichaetonotus australis* described from the east coast of Australia, is unique in that the large median dorsal spine precedes the lateral ones (the opposite is true for *Halichaetonotus sanctaeluciae* sp. n.). Moreover, the keel of the dorsal scales does not extend beyond the edge of the scales (Nicholas and Todaro 2005), whereas in *Halichaetonotus santaeluciae* sp. n. keels form a spiny process.

The new species also resembles *Halichaetonotus aculifer* (Gerlach, 1953) in terms of size and, most importantly, the shape of the hydrofoil scales. However, the presence of three long spines on the posterior trunk and the absence of ventral interciliary field scales in *Halichaetonotus sanctaeluciae* sp. n. are features that can easily differentiate this species from *Halichaetonotus aculifer* (see Gerlach 1953).

#### Halichaetonotus sp. 1

Fig. 1C

##### Material.

1 adult specimen, South Africa, KwaZulu-Natal, St Lucia beach, 22 February 2010, NAF Miranda legit.

##### Morphometry.

TL, 106.2 µm; PhL, 27.2 µm; PhIJ at U29.5; FuL, 21 (adhesive tube 17 µm); dorsal cuticular covering made up of seven columns of 17 overlapping, keeled scales. Scales round (up to 4 µm in diameter) on the head and neck region becoming ovoid (up to 8 × 4) over trunk. With exception of medial one, scales of posterior-most row bear keel extending into a 14 µm- long spine; 1+1 columns of hydrofoil scales ventrolaterally.

##### Remarks.

by virtue of the six spiny scales on the trunk rear, the animal appears different from any other species described so far. Unfortunately, a break of the slide occurred during the observation prevented the examination of the ventral side. Without detailed observations of the ventral scales’ shape, size and arrangement, we choose to avoid making a formal description of the species until additional specimens are observed.

#### Halichaetonotus sp. 2

Fig. 1D

##### Material.

2 adult specimens and 2 juveniles, South Africa, KwaZulu-Natal, St Lucia beach, 22 February 2010, NAF Miranda legit.

##### Morphometry.

TL, up to 104 µm; PhL, up to 25 µm; PhIJ at U29; FuL, up to 17.5 (adhesive tube 13 µm); dorsal cuticular covering made up of nine columns of 17 slightly overlapping, keeled scales. Scales round (up to 2–3 µm in diameter) on head and neck region becoming elliptical (up to 5 × 2 µm) over trunk. Ventrolaterally, 1+1 columns of hydrofoil scales bearing large lamellae.

##### Remarks.

Morphometry and general appearance of the specimens found at St Lucia match the metric and meristic characteristics of the cosmopolitan *Halichaetonotus decipiens* (Remane, 1929). Unfortunately, the detritus attached to the ventral side of one of the two adults and the large egg inside the second, prevented the observation of the cuticular details on the ventral side. Consequently, that identification to species cannot be made without reasonable doubts.

### Family Xenotrichulidae Remane, 1927. Genus Heteroxenotrichula Wilke, 1954

#### Heteroxenotrichula squamosa

Wilke, 1954

http://species-id.net/wiki/Heteroxenotrichula_squamosa

Fig. 1E

##### Material.

1 adult specimen, South Africa, KwaZulu-Natal, St Lucia beach, 22 February 2010, NAF Miranda legit.

##### Morphometry.

TL, 179 µm; PhL, 41.5 µm; PhIJ at U25.6; FuL, 52.3 µm (adhesive tube 20.5 µm); Dorsal cuticular covering made up of seven columns of 43–45 overlapping, scales extending laterally as hydrofoil scales. Ten flat scales on the inner margin of each furcal branch. Two pairs of head sensory cirri, 17–28 µm in length, 1 pair of head tentacles, 11.5 µm in length.

##### Remarks.

morphometry and general appearance of the specimen from St. Lucia is in general accordance with data reported for the cosmopolitan *Halichaetonotus squamosa*; in particular, the South African specimen appears of a size intermediate between individuals of the Mediterranean populations described by Wilke (1954) and Luporini et al. (1973) and specimens described from the Atlantic coast of France by Ruppert (1979).

### Genus Xenotrichula Remane, 1927

#### Xenotrichula sp.

http://species-id.net/wiki/Halichaetonotus

Fig. 1F

##### Material.

1 adult specimen. South Africa, KwaZulu-Natal, St Lucia beach, 22 February 2010, NAF Miranda legit.

##### Morphometry.

TL, 196 µm; PhL, 49.5 µm; PhIJ at U29; FuL, 27 µm (adhesive tube 10.2 µm); dorsal cuticular covering made up of 15 columns of 48 pedunculated scales (median column).

##### Remarks.

Morphometry and general appearance of the specimens found at St Lucia fall within the taxonomic range of the cosmopolitan *Xenotrichula intermedia* Remane, 1934 and of an as yet unnamed *Xenotrichula* sp. from Kuwait. The latter two species are siblings, sharing almost identical external morphology but very different organization of the muscular system (see Leasi and Todaro 2009). Technical reasons (several specimens are necessary) prevented the use of confocal laser scanning microscopy to study the organization of the muscular system in the only specimen available. Consequently, identification of the animal found at St. Lucia could not be made with sufficient confidence.

## Conclusion

The first report of gastrotrichs from South Africa shows that, despite the relatively low abundance of taxa retrieved so far, the potential for the discovery of new species unknown to science is remarkable. Soft-bodied organisms such as gastrotrichs must be processed fresh and live, in order to optimise observation of their key taxonomic characteristics. The unavoidable time delay (≥ 7 days) incurred between sample collections and analysis, due to the remote distance between the study site (St Lucia, South Africa) and the laboratory of analysis (Modena, Italy), has probably led to the loss of a number of potentially critical specimens in the samples (e.g. adult stages) and may account for the absence of gastrotrichs from some of the samples. It is, therefore, necessary to consider for the future the option of completing both sampling and analysis on site. This would also allow the collection of sufficient material to complement the traditional morphological studies with more advanced techniques, such as molecular analysis and confocal microscopy. This may prove invaluable towards the resolution of what is currently known in biogeography as the “meiofauna paradox” (Giere 2009). Indeed, it is necessary at this stage to ascertain whether the taxa that could not be conclusively identified to species level are actually already known cosmopolitan species, with broad geographic distribution, or rather new and localized species but “cryptic” in the sense that they cannot be distinguished only on the basis of external morphological characters. This will provide a valuable contribution towards the knowledge of the biodiversity of the St Lucia Estuary and the broader ecosystem of the World Heritage Site of which this wetland is an integral part.

## Supplementary Material

XML Treatment for Dactylopodola australiensis

XML Treatment for Pseudostomella

XML Treatment for Halichaetonotus sanctaeluciae

XML Treatment for Halichaetonotus

XML Treatment for Halichaetonotus

XML Treatment for Heteroxenotrichula squamosa

XML Treatment for Xenotrichula
